# Peptide-Based Anti-PCSK9 Vaccines - An Approach for Long-Term LDLc Management

**DOI:** 10.1371/journal.pone.0114469

**Published:** 2014-12-04

**Authors:** Gergana Galabova, Sylvia Brunner, Gabriele Winsauer, Claudia Juno, Bettina Wanko, Andreas Mairhofer, Petra Lührs, Achim Schneeberger, Arne von Bonin, Frank Mattner, Walter Schmidt, Guenther Staffler

**Affiliations:** AFFiRiS AG, Karl-Farkas-Gasse 22, Vienna, 1030, Austria; University of Catania, Italy

## Abstract

**Background:**

Low Density Lipoprotein (LDL) hypercholesterolemia, and its associated cardiovascular diseases, are some of the leading causes of death worldwide. The ability of proprotein convertase subtilisin/kexin 9 (PCSK9) to modulate circulating LDL cholesterol (LDLc) concentrations made it a very attractive target for LDLc management. To date, the most advanced approaches for PCSK9 inhibition are monoclonal antibody (mAb) therapies. Although shown to lower LDLc significantly, mAbs face functional limitations because of their relatively short *in vivo* half-lives necessitating frequent administration. Here, we evaluated the long-term efficacy and safety of PCSK9-specific active vaccines in different preclinical models.

**Methods and Finding:**

PCSK9 peptide-based vaccines were successfully selected by our proprietary technology. To test their efficacy, wild-type (wt) mice, *Ldlr* ^+/−^ mice, and rats were immunized with highly immunogenic vaccine candidates. Vaccines induced generation of high-affine PCSK9-specific antibodies in all species. Group mean total cholesterol (TC) concentration was reduced by up to 30%, and LDLc up to 50% in treated animals. Moreover, the PCSK9 vaccine-induced humoral immune response persisted for up to one year in mice, and reduced cholesterol levels significantly throughout the study. Finally, the vaccines were well tolerated in all species tested.

**Conclusions:**

Peptide-based anti-PCSK9 vaccines induce the generation of antibodies that are persistent, high-affine, and functional for up to one year. They are powerful and safe tools for long-term LDLc management, and thus may represent a novel therapeutic approach for the prevention and/or treatment of LDL hypercholesterolemia-related cardiovascular diseases in humans.

## Introduction

Hypercholesterolemia, in particular that of low density lipoprotein cholesterol (LDLc) is a major risk factor for the development of atherosclerosis and associated ischemic cardiovascular diseases. The main pathway for LDLc clearance from the blood circulation involves the low density lipoprotein receptor (LDLR) expressed on hepatocytes [1], [2]. Proprotein convertase subtilisin/kexin 9 (PCSK9), a ∼74 kDa protein synthesized in the liver interacts with LDLR [3], leading to its internalization and subsequent lysosomal degradation [3], [4], [5], [6], [7], thus downregulating the number of cell surface expressed LDLR molecules [8]. As a result, clearance of LDLc from the circulation by the LDLR is diminished, and propensity for cardiovascular disease is increased.

Genetic evidence for the role of PCSK9 as a major regulator of the cholesterol homeostasis [9] originates from studies showing that gain-of-function mutations are associated with decrease in the LDLR expression and LDLc internalization, while loss-of-function mutations are associated with increase in the LDLR surface expression and increased levels of LDL internalization [8]. Thus, beside genes encoding LDLR and apolipoprotein B (ApoB), the gene encoding PCSK9 constitutes a third locus involved in the development of the autosomal dominant hypercholesterolemia (ADH) [10].

Statins are the most commonly used drugs to treat hypercholesterolemia and result in profound LDLc reduction. However, many patients are unable to achieve their optimal lipid levels despite intensive statin therapy [11], [12]. Importantly, at the transcriptional levels statins up-regulate not only LDLR, but also PCSK9, causing the so-called paradox of statin treatment: although they induce a beneficial increase in LDLR, they also increase PCSK9, thus leading to LDLR degradation [13]. Thus, targeting PCSK9 alone or also in combination with statin treatment may offer a new avenue for a successful treatment of hypercholesterolemia [13]. Indeed, many studies confirmed the beneficial effect on LDLc levels by inhibiting the PCSK9-LDLR interaction. To date, the most advanced approaches for inhibiting PCSK9 action are monoclonal antibody (mAb) therapies [14], [15], [16], [17]. Several human mAbs were already evaluated in phase I and phase II clinical trials; these studies confirmed this approach as being beneficial and safe, not only as a monotherapy but also in combination with statins [15], [16], [17]. However, because of their relatively short *in vivo* half-lives, the use of mAbs faces functional limitations. Hence, long-term efficacy of mAb therapy is linked to frequent treatments and high costs. Active vaccination approaches could circumvent these drawbacks. The potential of active immunotherapeutic approaches for preventing from cardiovascular diseases and for cholesterol management is already known [18], [19], [20], [21].

In the current study, we describe an alternative approach for PCSK9 inhibition that provides the opportunity for long-term, safe LDLc cholesterol management, by using a peptide-based anti-PCSK9 active vaccination approach.

## Methods

Methods are described in detail in Text S1.

### Ethics statement

All animal experiments were performed in accordance with the guidelines for care and use of laboratory animals of the Austrian Animal Experiments Act. The protocol was approved and issued by the Vienna City Administration, Municipal Department 58, Legal affairs: Water Rights, Vienna, Austria (permit numbers: LF1-TVG-22/008-2009; M58/000504/2012/6; GZ: 134782/2013/13). At the end of each experiment mice were anaesthetized (using Rompun/Ketamidor; Bayer, Germany; Richter Pharma, Austria) and sacrificed according to the guidelines. In addition, experiments in Wistar rats (female, 6 weeks old), were performed by Aurigon Life Science GmbH (Tutzing, Germany) according to their guidelines and standard operating procedures (SOP).

### Animals

Inbred BALB/c and C57BL/6J mice (6–10 weeks old) were obtained from Charles River (Sulzfeld, Germany) and Janvier Labs (Saint Berthevin, France), homozygote *Ldlr* ^−/−^ knockout mice B6.129S7-*Ldlr^tm1Her^*/J from JAX mice (Bar Harbor, Maine, USA). Male *Ldlr* ^−/−^ mice were backcrossed to wt female C57BL/6J animals and first generation heterozygous mice were used for experimental procedures. Mice had access to food and water *ad libitum* and were kept under a 12 h light/dark cycle at the mouse facility of the Institute of Molecular Biotechnology of Austrian Academy of Science (IMBA), Vienna, Austria.

### Treatment and Immunization Scheme

Animals were immunized subcutaneously (s.c.) with 15 µg peptide antigen. Control animals received irrelevant peptide–KLH conjugates (peptide doses were adjusted to those used for AFFITOPE vaccines) formulated as described above. Prior to each immunization, 20 µl of blood sample was collected from the tail vein, and transferred into heparin tubes (BD), and plasma for further analysis was prepared according to the supplier. At the end of each experiment mice were anaesthetized (using Rompun/Ketamidor), according to the guidelines, and approximately 500 µl blood were collected from the retro-orbital venous plexus, and plasma was prepared. The experiments and sampling in Wistar rats were performed according to the guidelines and SOP of Aurigon Life Science GmbH. All plasma samples were stored at −20°C for further analysis. In general, either three or four immunizations (depending on the peptide) were performed in a biweekly interval, and short-term experiments were finalized 2 weeks after the last immunization (either 3^rd^ or 4^th^). For long-term experiments, animals were immunized three times in a biweekly interval and blood samples were collected prior to each immunization and monthly post 3^rd^ immunization. Re-boosting was tested either with one or two re-vaccinations.

### Vaccines

Peptide conjugates were adsorbed to 0.2% Alhydrogel (Brenntag Biosector, Denmark) and stored at 4°C. Vaccines were brought to room temperature (RT) and carefully mixed before each injection.

### Plasma Levels of Murine PCSK9 (muPCSK9) and Target Engagement

Plasma muPCSK9 concentration was determined by CircuLex mPCSK9 ELISA (CircuLex, Cy-8078, MBL), according to the manufacturer’s instructions.

### Plasma Total Cholesterol (TC) Measurement

Plasma samples were analyzed for the total cholesterol concentration (using the WAKO LabAssay Cholesterol Kit, Wako, Germany), according to the manufacturer’s instructions and absorbance was detected at 600 nm using a Sunrise microwell plate reader (Tecan, Switzerland).

### LDLR Sandwich ELISA

To determine the levels of LDLR in murine liver, 96-well Nunc-MaxiSorp plates were coated with goat polyclonal anti-LDLR (AF2255, R&D Systems). Plates were blocked with 1% BSA in PBS. Subsequently, liver lysates isolated as previously described [22] were incubated for 2 h at RT to capture the murine LDLR.

### Affinity Determination

Affinity parameters of vaccine-induced antibodies were analyzed by surface plasmon resonance (SPR), using a Biacore instrument (GE Healthcare).

### Inhibition of huPCSK9/huLDLR Interaction

The ability of AFFITOPE-induced antibodies to inhibit PCSK9-LDLR interaction was analyzed by SPR.

### Detection of T-cell Activation using ELISpot

Four 8–10 weeks old C57BL/6 mice per group were immunized four times in biweekly intervals. Splenocytes were isolated from each individual mouse and cell pools within each group were prepared. The mouse INF-γ cytokine assay (Mouse INF-γ ELISpot^PLUS^ Kit (ALP), Mabtech) was performed according to the manufacturer’s instructions. The amount of INF-γ producing cells were determined by using an (AID GmbH), with ELISpot 3.5 Software.

### Statistical Analysis

Data are given as means ±SEM. All values were evaluated for homoscedasticity and normality assumption using both Kolmogorov-Smirnov (KS) and Shapiro-Wilk normality tests. Comparisons between anti-PCSK9 vaccinated animals and the related negative controls were assessed by by one-way ANOVA test with subsequent Tukey’s multiple comparison test, by unpaired two-tailed Student’s *t*-test, and by two-way ANOVA test, followed by Bonferroni multiple comparisons test. A *p-*value of ≤0.05 was considered statistically significant.

## Results

### Selection and Immunogenicity of anti-PCSK9 Vaccines in wt Mice

To be effective, an anti-PCSK9 vaccine should ultimately prevent the binding of PCSK9 to the LDLR. Using proprietary technology [23], short peptides (8–13 amino acids in length), mimicking parts of the N-terminus of the mature human PCSK9 protein responsible for the interaction with human LDLR were selected and described (Patent application PCT/EP2013/067797; WO 2014/033158). The peptides were conjugated to the carrier protein KLH, and subsequently formulated with 0.2% Alhydrogel. Importantly, since the PCSK9 regions to be targeted are almost identical between human, mice and rats, the antibodies generated are expected not only to prevent the interaction of huPCSK9 with the huLDLR, but also to block this interaction in rodents.

**Figure 1:**
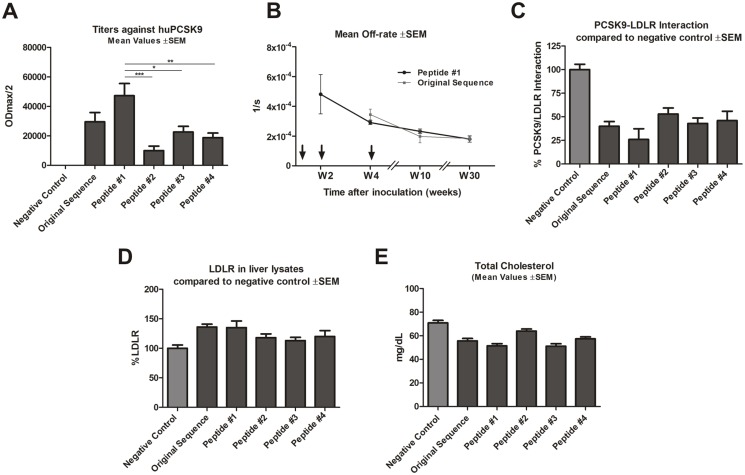
Anti-PCSK9 vaccine efficacy in BALB/c mice. (**A**) Antibody titers (ODmax/2) against huPCSK9 protein, for sera generated from different peptides generated upon 4 vaccinations in biweekly intervals. Values are means ±SEM (n = 8/group). Significance values relative to Peptide #1 (*p<0.05; **p<0.005, ***p<0.001) were obtained by one-way ANOVA test with subsequent Tukey’s multiple comparisons test. (**B**) Mean off-rate values ±SEM of antibodies generated upon three subsequent immunizations (marked by arrows) with Peptide #1 and original PCSK9 sequence in a biweekly interval: W0, W2 and W4. (**C**) PCSK9-LDLR binding affinities in the presence of anti-PCSK9 antibodies, generated from different peptides. Values are means ±SEM, shown as a percentage of that for the negative control. (**D**) LDLR levels in liver hepatocytes from mice inoculated with different peptides, shown as a percentage of the negative control. Pooled liver lysates within each group (n = 5/group) were analyzed; error bars represent mean values ±SEM of duplicate measurements. (**E**) Mean values of TC levels (mg/dL) for mice inoculated with different peptides. Values are means; error bars represent ±SEM. Samples for (**A**), (**C**), (**D**) and (**E**) taken at week 8. W: week.

Vaccines tested in wt mice were found to elicit an antibody response to both human (**Figure 1A**) and mouse PCSK9 protein (data not shown). In addition, AFFITOPE-based vaccines (termed Peptides #1, #2 etc.) were compared to a vaccine that contains the PCSK9-derived target peptide sequence (termed “original sequence”) as antigen. As shown in **Figure 1A** the newly-selected vaccine generated by Peptide #1 elicited an antibody response against PCSK9 that was over 1.6-fold greater than that evoked by the original sequence peptide. Importantly, Peptide #1 elicited significantly higher titers in comparison to Peptides #2; #3 and #4.

In addition, SPR analysis showed a similar steadily decrease of off-rate values of induced antibodies after each immunization with Peptide #1 and original sequence, indicating that vaccine-induced antibodies increased their affinity with each injection and underwent affinity maturation (**Figures 1B**).

To evaluate the functional abilities of peptide-induced antibodies to inhibit the huPCSK9-huLDLR interaction, inhibition studies using SPR analysis were performed. All induced sera interfered with this interaction (**Figure 1C**). However, sera derived from animals treated with Peptide #1 reduced huLDLR-huPCSK9 binding by 70%, whereas those derived from mice treated with original sequence reduced huLDLR-huPCSK9 binding by 50% (**Figure 1C**), compared to the control. As the induced antibodies block the huPCSK9-huLDLR interaction *in vitro*, and since antibodies effectively cross-react with mouse and rat PCSK9, reduction of this interaction *in vivo* is also expected, which one would result in increased LDLR expression and reduced TC levels. To test this, liver LDLR levels in hepatocytes of immunized mice were analyzed, and again Peptide #1 showed the strongest effect in comparison to the other selected peptides. It raised the LDLR levels up to 140%, which was similar to the level induced by the original peptide vaccine (**Figure 1D**). Finally, upon immunization with Peptide #1, TC levels were significantly reduced (**Figure 1E**) in comparison to the control peptide, confirming their functional abilities *in vivo* and highlighting Peptide #1 as the optimal candidate for an anti-PCSK9 vaccine approach. Based on all performed evlaluations (**Figure 1**) Peptide #1 was selected and further evaluated in additional animal models and long-term experiments.

### Efficacy of anti-PCSK9 Vaccine in Wistar Rats

**Figure 2:**
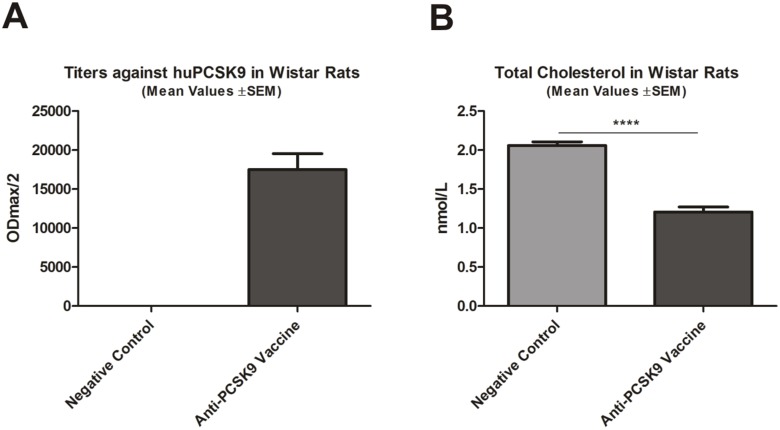
Anti-PCSK9 vaccine efficacy in Wistar rats. (**A**) Titers (ODmax/2) against huPCSK9 (n = 8/group) generated upon 4 vaccinations in biweekly intervals. (**B**) TC levels in treated animals (nmol/L), compared to control. Bars and error bars represent mean values ±SEM; significance compared to control values (****p<0.0001) was obtained by a 2-tailed Student’s *t-*test.

In order to confirm the immunogenicity and functionality of the anti-PCSK9 vaccine in an alternative animal species, in a first step Wistar rats were treated with this vaccine. As depicted in **Figure 2A**, the vaccine induced high titers against human and rat PCSK9 (data not shown). This induction of the PCSK9-specific antibody response was paralleled by a significant decrease of TC (compared to control; ****p<0.0001) (**Figure 2B**).

### Vaccine-Induced Antibodies are Engaging PCSK9 *in vivo*

**Figure 3:**
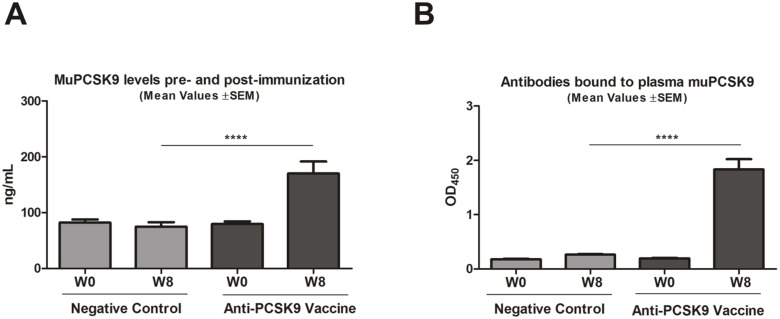
Vaccine-generated antibodies target plasma muPCSK9. (**A**) muPCSK9 levels in plasma samples from pre-immunized (W0) and post-immunized mice (W8). Bars represent mean levels of detected muPCSK9 levels in plasma samples, error bars represent ±SEM, (n = 10 mice/group). Significance values were calculated by two-way ANOVA, followed by Bonferroni multiple comparisons test (n = 10 mice/group) (***p<0.001). (**B**) Direct detection of antibodies bound to plasma muPCSK9 in plasma samples from pre-immunized (W0) and post-immunized mice (W8). Increased OD_450_ is indicative for anti-PCSK9-vaccine induced antibodies directly binding to muPCSK9. Bars represent mean values, error bars represent ±SEM, (n = 10 mice/group). Significance values were calculated by two-way ANOVA test with subsequent Bonferroni multiple comparisons test, (***p<0.001). W: week.

Although, the reactivity of vaccine-induced antibodies against PCSK9 protein was shown (**Figures 1A** **,** **2A**) evidence for a direct interaction between these antibodies with the mouse target protein (muPCSK9) *in vivo* is missing. Importantly, studies report that anti-PCSK9 antibody treatment raises muPCSK9 plasma levels. This phenomenon was explained by the stabilization of the protein by antibodies, leading to a delayed clearance of the protein and provides a hint for direct target engagement *in vivo* [22]. Consequently, an increase of PCSK9 protein concentration in plasma in vaccine-treated animals was expected. Therefore, prior to the first vaccination (week 0, W0) and two weeks after the last vaccination (week 8, W8) plasma levels of muPCSK9 were measured. The related control group was vaccinated with an irrelevant peptide. As expected, before treatment (week 0), in all animals plasma muPCSK9 levels were comparable (**Figure 3A**) and did not change over the time upon treatment with the negative control vaccine. In contrast, all anti-PCSK9 vaccine treated animals (W8) showed a significant elevation in muPCSK9 plasma levels (***p<0.001), indicative of *in vivo* target engagement (**Figure 3A**). These results were in line with the amount of PCSK9-specific serum antibodies in treated animals (**Figure 1A**). Importantly, despite a higher amount of circulating PCSK9, the TC levels were decreased. For further evidence of target engagement, an additional ELISA-based assay, detecting murine antibodies directly bound to muPCSK9, was performed. For this purpose, muPCSK9 originating from plasma samples of pre-immunized (W0) and immunized animals (W8) was captured onto ELISA plates, and vaccine-induced PCSK9-bound murine antibodies were detected using an anti-mouse IgG antibody. The significantly higher OD_450_ signal depicted in **Figure 3B** in anti-PCSK9 vaccine-evoked plasma confirmed a direct binding of vaccine-induced antibodies to the target protein (PCSK9). In summary, the selected anti-PCSK9 vaccine generates specific antibodies that directly target and bind muPCSK9 in the blood circulation.

### PCSK9 Vaccine and Long-term LDLc Management

**Figure 4:**
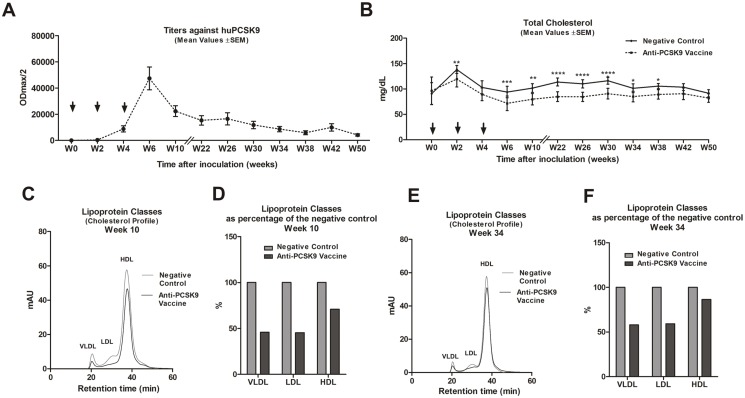
Long-term LDL cholesterol management upon anti-PCSK9 vaccination. (**A**) Long-term evaluation over 50 weeks post prime immunization of titers against huPCSK9 (ODmax/2), generated upon 3 vaccinations in a biweekly interval (marked by arrows). Values are means ±SEM, n = 10 mice/group. (**B**) Measurement of TC values over the same time period as (A). Values are means; error bars represent ±SEMs. Significance values were calculated by comparison with the control group, by two-way ANOVA test, followed by Bonferroni multiple comparisons test, at the indicated time-points (n = 10 mice/group). Significance values are indicated as follows: *p<0.05; **p<0.01; ***p<0.001; ****p<0.0001. (**C**) FPLC lipoprotein profile of pooled plasmas within the groups (n = 10), 10 weeks post prime immunization. (**D**) Plasma lipoprotein levels, for three classes (VLDL, LDL, HDL), 10 weeks after prime immunization, expressed as a percentage of that for the negative control. (**E**) FPLC cholesterol lipoprotein profiles (VLDL, LDL and HDL) of pooled plasmas within the groups (n = 10), 34 weeks post prime immunization. (**F**) Plasma lipoprotein levels, for three classes (VLDL, LDL, HDL), 34 weeks after prime immunization, expressed as a percentage of that for the negative control. W: week.

PCSK9-specific immunotherapy using mAbs revealed a clear reduction in LDL cholesterol [24]. However, those approaches are limited by their short-term effects. As pointed out above, we confirmed the efficacy of anti-PCSK9 approach after primary immunizations (**Figure 1** **and** **2**). However, these results did not address the question of the persistence of the effect, therefore, we undertook longer-term studies to assess this. BALB/c mice were vaccinated three times in a biweekly interval and titers and TC levels were monitored for up to 12 months post prime immunization (**Figure 4**). As expected, six to ten weeks after the prime immunization anti-huPCSK9 titers reached their peak levels (ODmax/2 = 50.000) (**Figure 4A**) and simultaneously TC decreased significantly (W6, *p*<0.001; W10, *p*<0.01; W22, W26 and W30, *p*<0.0001) in the PCSK9 vaccine-treated animals compared to the control group (**Figure 4B**). Subsequently, the amount of plasma anti-PCSK9 antibodies started to decrease with an *in vivo* half-life of about 4 months (**Figure 4A**). This was in line with the stepwise changes in the TC levels, which lost its significance (compared to control group) at 42–50 weeks after prime immunization (**Figure 4B**). In addition, particular time points (10 and 34 weeks post prime immunization) were selected for detailed cholesterol lipoprotein profile analysis (by fast protein liquid chromatography, FPLC) to measure VLDL, LDL and HDL levels. It is known that wt mice contain very low amounts of ApoB-containing lipoproteins (VLDL and LDL). These carry the majority of circulating cholesterol in HDL particles. Importantly, mouse HDL contains ApoE, which makes its binding to LDLR possible [25], [26]. Therefore, as expected anti-PCSK9 vaccination in wt mice resulted in a lowering of VLDL, LDL, and HDL levels (**Figure 4C–F**). In particular, 10 weeks after prime immunization the decrease in LDL was as much as 50% compared to the control group (**Figure 4D**). In contrast, 34 weeks post prime immunization the LDL decrease was less pronounced: by about 35–40% (**Figure 4F**)**.**

### Reactivation of the Humoral Immune Response after Booster Immunization

**Figure 5:**
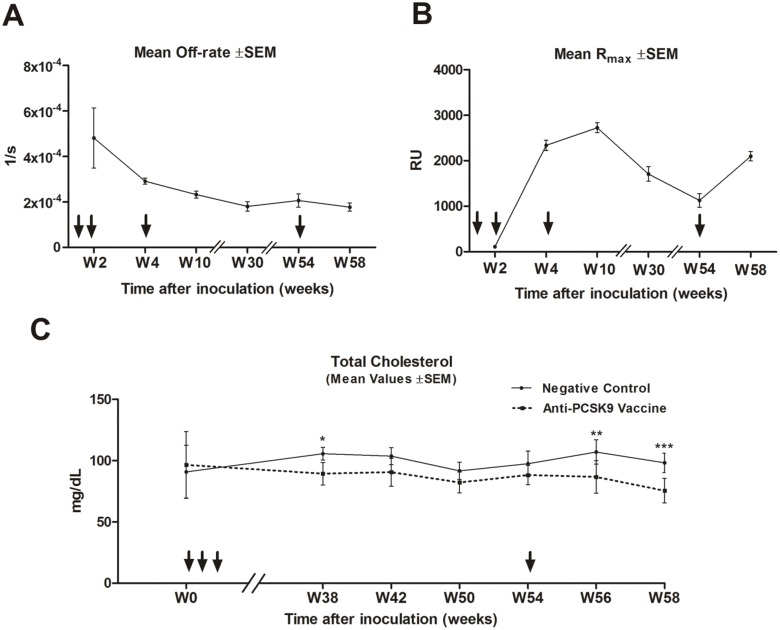
Anti-PCSK9 vaccine approach is boostable, and induced functional antibodies even after a single yearly re-boost. (**A**) Off-rate values generated upon 3 vaccinations (marked with arrows) in a biweekly time period in BALB/c mice, followed by a single re-boost in week 54 (marked with an arrow), for samples (n = 10) taken at weeks 2, 4, 10, 30, 54 and 58. Values shown are means ±SEM. (**B**) R_max_ values (n = 10) for the vaccination timecourse shown in (A). Values shown are means ±SEM. Note the re-boost effect seen at week 58 on the amount of antibodies (R_max_ values) upon single re-vaccination in week 54. (**C**) Total cholesterol values for PCSK9-inoculated and control mice during a 58-week timecourse. Values are means ±SEM. Significance values were calculated by comparison with the control group, using a two-way ANOVA test with subsequent Bonferroni multiple comparisons test, at the indicated time-points (n = 10 mice/group). Significance values are indicated as follows: *p<0.05; **p<0.01; ***p<0.001. Note the slight loss of effect on TC levels over time (for example, by W42 and W50), and the induction of a significantly beneficial decrease following the re-boost immunization at W54. W: week.

Since at the end of the long-term study the levels and functionality of vaccine-induced antibodies were declining (**Figure 4**), the question rose whether the anti-PCSK9 antibody response could be reactivated upon booster immunization. To test this, 12 months post prime immunization re-vaccinations with the same dose were performed, and plasma antibody concentrations and TC were analyzed (**Figure 5**). SPR analysis, to define off-rate values and R_max_ values, confirmed the successful reactivation of high-affinity antibody production upon a single booster vaccination in W54 (**Figure 5A and 5B**). In parallel, plasma TC levels decreased to levels similar to those shortly after the primary immunizations (W56, *p*<0.01; W58, *p*<0.001) (**Figure 5C**). Altogether, these data revealed the potential of a yearly schedule of re-boosting immunization.

### Anti-PCSK9 Vaccine Strongly Reduces LDLc in *Ldlr* ^+/−^ Mice

**Figure 6:**
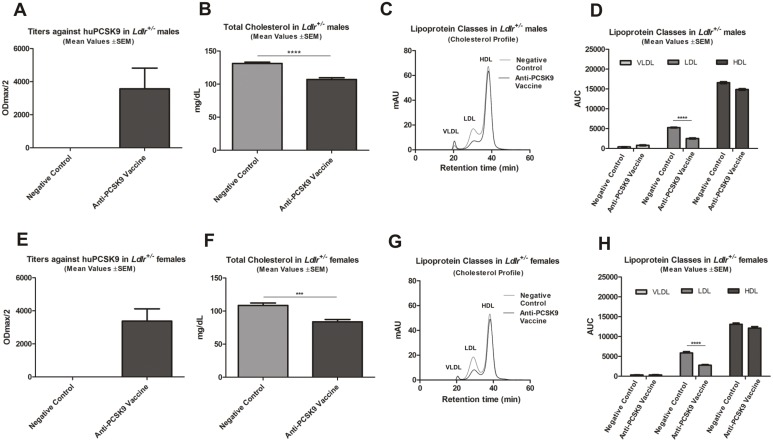
Anti-PCSK9 vaccine significantly lowers the LDLc in *Ldlr* ^+/−^ male and female mice upon 4 immunizations. (**A**) Mean titers (ODmax/2) ±SEM against huPCSK9 (n = 9). (**B**) TC levels (mg/dL), bars and error bars represent mean values (n = 9) ±SEM. (**C**) FPLC cholesterol lipoprotein profile (VLDL, LDL and HDL) of pooled plasmas from immunized mice (n = 9) and their related controls. (**D**) Mean values of the area under the curve (AUC) ±SEM evaluated by FPLC cholesterol lipoprotein profile analysis of the VLDL, LDL and HDL in immunized male mice and their related controls (n = 9). Significance values were calculated by comparison with control values for LDLc (****p<0.0001) using a 2-tailed Student’s *t-*test. (**E**) Mean titers (ODmax/2) ±SEM against huPCSK9 (n = 9). (**F**) TC levels (mg/dL), bars and error bars represent mean values (n = 9) ±SEM. (**G**) FPLC cholesterol lipoprotein profile (VLDL, LDL and HDL) of pooled plasmas (n = 9). (**H**) Mean AUC values ±SEM, evaluated by FPLC cholesterol lipoprotein profile analysis of VLDL, LDL and HDL cholesterol in immunized female mice and their related controls (n = 9). Significance values were calculated by comparison with negative control values, using a 2-tailed Student’s *t-*test. Significance values are indicated as follows: ****p<0.0001. All samples taken at week 8.

Since LDLc levels are low in wt mice, the effect of the anti-PCSK9 vaccine on lowering LDLc was evaluated in an independent LDLR-heterozygous mouse model. It is known that elimination of functional LDLR in mice (in a knock-out transgenic strain) elevates LDLc levels [2]. However, considering that our approach targets the PCSK9-LDLR interaction, the presence of at least one allele of the receptor is essential in order to evaluate the effect of the anti-PCSK9 vaccine on the LDLR and thus on LDLc. Therefore, *Ldlr* ^+/−^ mice (also showing elevated LDLc levels) were used for further experiments (**Figure 6**). Previous studies reported a difference in the lipoprotein profile between males and females, which can be attributed to the reproductive cycle phases of the females [2]. Thus *Ldlr* ^+/−^ female and male mice were immunized. As expected, the immunizations led to the generation of anti-huPCSK9 titers (**Figure 6A, E**) in both genders. In parallel, a significant reduction in TC levels in comparison to their negative control groups was observed (**Figure 6B, F**; *p*<0.0001; *p* = 0.0002)**.** Importantly, FPLC analysis revealed a significant (*p*<0.0001) reduction of the LDLc (**Figure 6C–D, G–H**), compared to the negative control. Interestingly, the mainly LDL class of lipoproteins was reduced by more than 50% (**Figure 6C–D, G–H**), again confirming the use of anti-PCSK9 vaccine as a powerful approach for long-term LDLc management.

### Anti-PCSK9 vaccine and pre-clinical safety evaluations

For vaccinations against self-proteins, different safety prerequisites have to be considered, such as absence of target-specific T cell response and absence of cross-reactivity of induced antibodies with other endogenous proteins.

**Figure 7:**
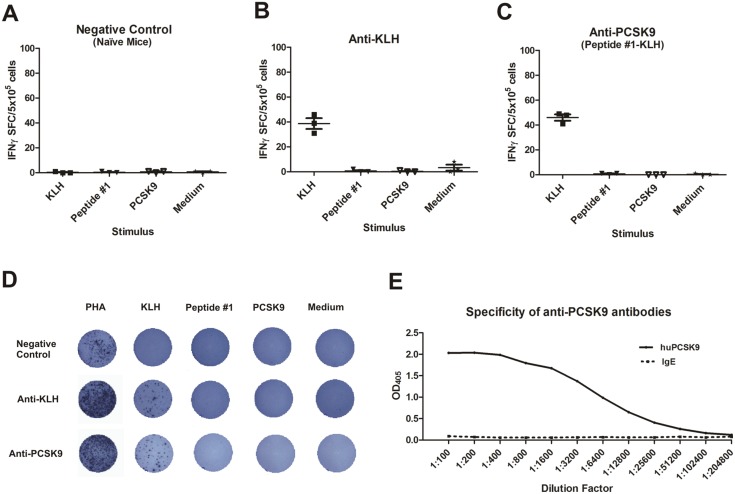
Anti-PCSK9 vaccination does not activate PCSK9-specific T cells. ELISpot analysis for INF-γ-releasing splenocytes isolated from C57BL/6 (H-2D^b^) mice. (**A**) naϊve mice (negative control). (**B**) mice immunized with KLH alone. (**C**) mice immunized with anti-PCSK9 vaccine. Triplicate measurements were performed from cell pools within the group. (**D**) Representative pictures from ELISpot assay are shown, including PHA stimulation as positive control. (**E**) ELISA analysis of anti-PCSK9 vaccine-induced antibodies against PCSK9 and IgE. The antibodies generated do not cross-react with IgE.

To exclude peptide antigens that could potentially lead to the formation of PCSK9-specific T cells (T helper as well as cytotoxic T cells), in a first step, we performed an *in silico* analysis of selected peptide antigens using different online available T cell epitope prediction algorithms (SYFPEITHI, IEDB Analysis Resource). Neither a human nor a clear mouse T cell epitope (for both MHC class I and II) was predicted. Only the SYFPEITHI *in silico* evaluation revealed a potential MHC class I molecule binder in mice carrying the H-2D^b^ allele. Hence, we tested whether the anti-PCSK9 vaccine has the capacity to induce cytotoxic T cells *in vivo* in C57BL/6 (H-2D^b^) mice. Animals were either immunized with anti-PCSK9 peptide vaccine, with KLH alone, or left untreated, and T cell activation was evaluated using an ELISpot assay. As depicted in **Figure 7A–D**, the *ex vivo* re-stimulation of splenocytes isolated from immunized mice with the peptide used for immunization or with PCSK9 protein did not result in the induction of INF-γ-releasing T cells. In fact, only the KLH re-stimulation or the stimulation with the mitogen phytohemagglutinin (PHA) as positive control resulted in INF-γ release (**Figure 7D**). Thus, the selected peptide used for immunization does not constitute a T cell epitope leading to the induction of PCSK9 specific T cells.

In order to evaluate potential cross-reactivity of induced antibodies to other human proteins, BLAST searches were performed. These analyses confirmed that the selected peptide does not share homology to any other human protein. However, Peptide #1 appeared to have 50% sequence identity with a stretch of amino acids from IgE. But as can be seen in **Figure 7E**, vaccine-induced antibodies did not cross-react with IgE protein.

Furthermore, the specificity of these antibodies was evaluated using a ProtoArray human protein chip. Vaccine-induced anti-PCSK9 antibodies did not cross-react with more than 9000 human proteins spotted on the array, indicating that the induced humoral immune response is indeed very specific for PCSK9. Only one human protein, microfibrillar-associated protein 1 (MFAP1) was recognized (data not shown). However, subsequent BLAST analysis comparing the sequences of the Peptide #1 and MFAP1, revealed a lack of sequence homology. Importantly, and in line with our BLAST analysis, double immunofluorescence analysis using Peptide #1-induced sera and MFAP1-specific polyclonal Abs on frozen human tissue arrays revealed an absence of co-localization of the corresponding signals (**Figure S1**), indicating that vaccine-induced antibodies do not cross-react with MFAP1 in human tissue.

Importantly, there was no evidence of systemic toxicity resulting from vaccinations. Local reactions in the expected range were noted at the injection site. Thus besides being effective in the longer-term, anti-PCSK9 vaccines were well tolerated.

Finally, modified-SHIRPA tests were performed in order to identify potential side effects of the vaccines on muscle, sensory, and cerebellar functions. Vaccines were well tolerated in the animals tested, since immunized mice were healthy and displayed normal sensory and motor functions (**Figure S2**).

Altogether, the current data validate the peptide-based anti-PCSK9 vaccine as highly specific and safe.

## Discussion

This first study of PCSK9 inhibition using an active peptide-based anti-PCSK9 vaccination approach in animals demonstrates that a sustained TC as well as LDLc reduction can be achieved for up to one year. The short peptides used as antigens differ in their amino acid sequence from the native sequence to be targeted, but still have the capacity to induce an antibody response highly specific for PCSK9. As selected peptides also do not exhibit sequence identity with other human proteins, they are “foreign” to the human immune system and, thus, these peptide vaccines in general are a potent means of inducing antibodies to self-antigens [23]. The PCSK9-specific vaccines described here generate antibodies that not only recognize huPCSK9 but also efficiently interact and block the PCSK9 protein action in mice and rats. Thus, these vaccines, designed to be effective in man, can be tested in proof-of-concept studies using rodents.

As depicted in **Figure 1** **–** **2**, peptide-based vaccines are highly immunogenic, generate specific and functional antibodies able to bind PCSK9 and inhibit its interaction with LDLR. Using SPR analysis R_max_ values (a combined measure for antibody concentration and affinity) as well as off-rate values (beside the on-rate value the second affinity determining parameter) have been analyzed. R_max_ values were strongly increasing and off-rate values of induced antibodies were decreasing significantly during the course of immunizations (**Figure 1B** **and** **Figure 5A–B**), indicating the affinity maturation of induced antibodies.

It is known that inhibition of the PCSK9-LDLR interaction leads to up-regulation of LDLR in hepatocytes, thus promoting plasma cholesterol uptake from the blood circulation [21] [22]. In line with these observations, our approach inhibited the PCSK9-LDLR interaction which consequently resulted in LDLR up-regulation. This resulted in significantly decreased TC levels and in particular LDLc compared to the negative control group. Although, the Peptide #1-induced PCSK9 specific antibody response was significantly higher compared to the one elicited by other tested PCSK9 vaccines, the effect on TC reduction did not significantly differ between PCSK9-vaccine treated groups (**Figure 1**). This phenomenon can be explained by a saturation effect: upon a certain plasma antibody concentration all circulating PCSK9 proteins are occupied and thus exceeding antibodies have limited effect on TC directly after prime immunization. However, high titers after prime immunization prolong the time period in which the antibody levels are high enough to sufficiently block PCSK9 function in plasma and thus this may influence cholesterol management on the long-term. In comparison to previously described studies [22] we observed a sustained presence of functional antibodies able to reduce TC and LDLc for up to one year (**Figure 4** **–** **5**). The half-life time of vaccine-generated anti-PCSK9 antibodies was about four months (**Figure 4**), exceeding by far the half-life time of passively transferred antibodies. In fact, for a sustained effect on LDLc lowering repeated applications of anti-PCSK9 mAbs were necessary [14], [15], [16], [17], [22] confirming that the limiting factor for mAb treatment is their short half-lives.

Since cholesterol management in humans needs to last for years, fading vaccine-induced immune responses have to be reactivated after certain time frames. Thus, booster experiments one year after prime immunizations were performed. Re-vaccination successfully reactivated the anti-PCSK9 antibody response in treated mice, not only in terms of the amount of antibodies, but also in terms of generation of high-affinity and functional antibodies, able to reduce TC plasma levels (**Figure 5**).

It is known that LDLc levels are low in wt mice, and that the primary circulating lipoprotein is HDL that contains ApoE, which makes the binding of HDL particles to LDLR possible. The anti-PCSK9 antibodies target and inhibit the PCSK9-LDLR interaction (**Figure 1C**), and subsequently upregulate LDLR (**Figure 1D**) thereby influencing the plasma levels of all ligands able to bind LDLR. This explains the reduction of HDLc and thus TC in wt mice upon anti-PCSK9 immunization. In contrast, the vast majority of HDL particles in humans do not contain ApoE and therefore do not bind to LDLR. Thus the effect of blocking the PCSK9-LDLR interaction by vaccine-induced antibodies in humans will result mainly in LDLc lowering.

Since LDLc levels are low in wt mice, the effect of the anti-PCSK9 vaccine to lower LDLc was also tested in *Ldlr* ^+/−^ mice, a suitable model for this purpose, due to their elevated plasma LDLc. Importantly, upon immunization, LDLc levels significantly decreased by 50–55% in comparison to control (**Figure 6**). Altogether, the data obtained from the *Ldlr* ^+/−^ mice provided additional confirmation of the powerful effect of the peptide-based anti-PCSK9 vaccine, in particular on LDLc.

The aim of therapeutic vaccines targeting self-proteins is to obtain antibody titers that effectively and specifically neutralize the protein of interest without concomitantly inducing a target-specific T cell response. Accumulation of auto-reactive T cells can induce irreversible tissue damage, with potentially life-threatening consequences [27]. To avoid the induction of target specific T cells but still to be able to induce an effective B cell response short peptide sequences were coupled to the foreign carrier protein KLH that provides T helper cell epitopes. Hence for the development of a B cell vaccine targeting PCSK9, we followed the strategy to circumvent T cell tolerance rather than break it. Furthermore, the selected peptide antigens are shorter (10 amino acids) than peptides known to bind MHC class II molecules. This was also confirmed by *in silico* analysis. In contrast, selected peptide antigens are long enough to potentially serve as MHC class I binders. *In silico* analysis highlighted Peptide #1 as a potential MHC class I binder in animals carrying the H-2D^b^ allele. However, T cell activation analysis using ELISpot provided evidence that immunization using Peptide #1 does not induce cytotoxic T cells *in vivo* (**Figure 7A–D**), confirming Peptide #1 as an exclusive B cell antigen. Furthermore, the reversibility of PCSK9 specific antibody response was evaluated in long term *in vivo* experiments (**Figure 4** **–** **5**). The data indicate that antibody production is reversible and thus is exclusively guided and controlled by the administration of peptide vaccines (“exogenous stimulus”), and not by endogenous PCSK9.

The lack of cross-reactivity of the anti-PCSK9 vaccine-induced antibodies towards more than 9000 tested human proteins using the ProtoArray technology, including the IgE protein (shown to have 50% sequence identity to the antigenic peptide) further proved the specificity of induced antibodies (**Figure 7E**, data not shown). Although, ProtoArray analysis revealed binding of vaccine-induced antibodies to MFAP1, such reactivity on human frozen tissue sections could not be detected (**Figure S1**), confirming the specificity of Peptide #1-induced antibodies for PCSK9. Interestingly, human, mouse, and rat MFAP1 proteins have 100% sequence homology. Since no side effects (**Figure S2**), either in mice or in rats have been observed after immunization with Peptide #1, such reactivity towards MFAP1 is not expected to take place *in vivo*. The clinical assessment of PCSK9-treated animals (**Figure S2**) did not provide evidence of an autoimmune reaction. Immunizations either in short or long-term experiments had no side effects in terms of weight loss, kidney failure and pathological changes in different organs or mortality in mice. Furthermore, anti-PCSK9 vaccine was well tolerated and safe in rats, guinea pigs, hamsters and rabbits, as demonstrated by patho-histological analysis and analysis of the blood haematology and blood chemistry (data not shown). Altogether, this confirms the anti-PCSK9 vaccine approach as highly specific and safe.

In summary, by using the proprietary AFFITOME technology, we successfully selected antigenic peptides that mimic original PCSK9 epitopes and upon immunization induce long-lasting, high-affinity and functional PCSK9-specific antibodies in experimental animals. This leads to significant reduction in LDL cholesterol for up to one year. Thus, the peptide-based anti-PCSK9 vaccination is an innovative approach and a powerful strategy for the long-term management of LDLc levels.

### Clinical perspectives

To date, several PCSK9-inhibiting approaches have been proposed, including gene silencing and inhibition of PCSK9-LDLR binding by monoclonal antibodies, small peptides or adnectins. Currently the most advanced PCSK9 inhibitors (in phase III studies), achieving LDLc reductions of up to 65–70%, are the monoclonal antibodies, either in combination with, or without statin therapy. Importantly, the effects of these were greatest upon frequent administration, at either two- or four-week intervals, making the cost/benefit ratio a very important issue. The present study describes the preclinical development of a PCSK9-targeting active vaccine approach. All vaccine candidates, selected based on the mechanism of molecular mimicry, were found to elicit PCSK9-specific antibodies that reduce beneficially LDLc levels up to 50–55% when applied to experimental animals. The advantage of the sustained LDLc decrease, which lasted for up to one year, demonstrates this approach as being a promising strategy for long-term cholesterol management. Finally, these data provides the basis for an early clinical testing of the most compelling candidate(s).

## Supporting Information

Figure S1 **Vaccine-induced anti-PCSK9 antibodies do not cross-react with MFAP1 in the extracellular matrix.** Double immunofluorescence analysis of human kidney tissue cryosection shows the expression of PCSK9 in the kidney tubular epithelium (green/asterisks) and the expression of MFAP1 in the extracellular matrix (red/arrows). Note the lack of co-localization between plasma anti-PCSK9 antibodies generated upon immunization with Peptide #1 (green) and MFAP1 (red). DAPI (blue) was used as a counterstain.
(TIF)

Figure S2 **Mice immunized with anti-PCSK9 vaccine are healthy with normal motor and sensory function.** **(A)** Body weight of mice in grams (g). **(B)** General health status of mice immunized with anti-PCSK9 vaccine in comparison to the negative control. **(C)** Motor abilities of mice immunized with anti-PCSK9 vaccine in comparison to the negative control group. **(D)** Sensory function of mice immunized with anti-PCSK9 vaccine in comparison to the negative control group. **(E)** Handling behavior. Evaluations were performed according to the modified SHIRPA test. Bars and error bars represent mean values (n = 5 mice/group) ±SEM.
(TIF)

Text S1 **Methods.**
(DOCX)
