# Supplementary figures and images for: Peptide-Based Anti-PCSK9 Vaccines - An Approach for Long-Term LDLc Management
Source: PLoS One. 2014 Dec 4;9(12):e114469. doi: 10.1371/journal.pone.0114469 (PMC4256444; doi:10.1371/journal.pone.0114469)

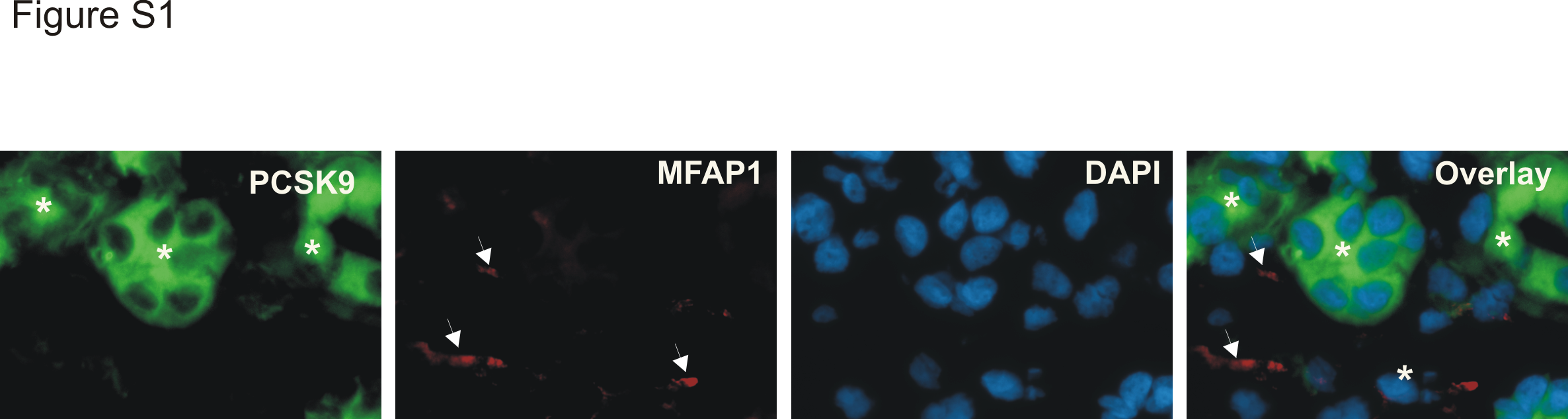

Supplement: Figure S1 — Vaccine-induced anti-PCSK9 antibodies do not cross-react with MFAP1 in the extracellular matrix. Double immunofluorescence analysis of human kidney tissue cryosection shows the expression of PCSK9 in the kidney tubular epithelium (green/asterisks) and the expression of MFAP1 in the extracellular matrix (red/arrows). Note the lack of co-localization between plasma anti-PCSK9 antibodies generated upon immunization with Peptide #1 (green) and MFAP1 (red). DAPI (blue) was used as a counterstain. (TIF) [file pone.0114469.s001.tif]

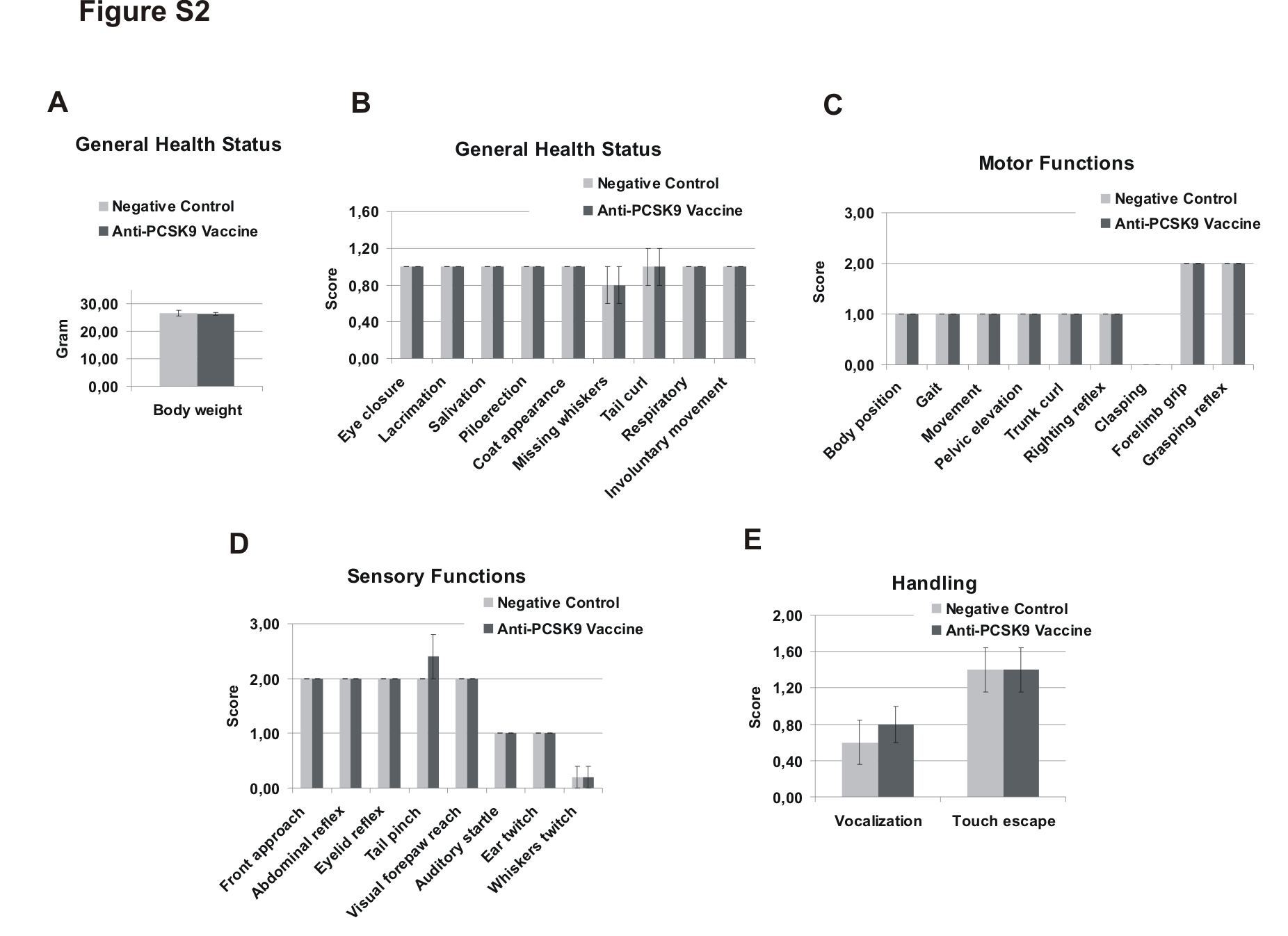

Supplement: Figure S2 — Mice immunized with anti-PCSK9 vaccine are healthy with normal motor and sensory function. (A) Body weight of mice in grams (g). (B) General health status of mice immunized with anti-PCSK9 vaccine in comparison to the negative control. (C) Motor abilities of mice immunized with anti-PCSK9 vaccine in comparison to the negative control group. (D) Sensory function of mice immunized with anti-PCSK9 vaccine in comparison to the negative control group. (E) Handling behavior. Evaluations were performed according to the modified SHIRPA test. Bars and error bars represent mean values (n = 5 mice/group) ±SEM. (TIF) [file pone.0114469.s002.tif]
