# Supplementary material for: Peptide-Based Anti-PCSK9 Vaccines - An Approach for Long-Term LDLc Management
Source: PLoS One. 2014 Dec 4;9(12):e114469. doi: 10.1371/journal.pone.0114469 (PMC4256444; doi:10.1371/journal.pone.0114469)
Supplement: Text S1 — Methods. (DOCX) [file pone.0114469.s003.docx]

**Text S1**

**Methods**

**AFFITOPEs^®^ and Peptides**

Using the proprietary AFFITOME^®^ technology, short peptides termed AFFITOPEs were selected, which mimic parts of the native human sequence or structure of the human PCSK9 protein that are responsible for the interaction with human LDLR. AFFITOPES^®^ and control peptides were synthesized at EMC microcollections (Tübingen, Germany) using conventional Fmoc chemistry. Subsequently, peptides were purified by HPLC reaching a purity grade >95%. To enable coupling of the AFFITOPES**^®^** to the carrier proteins such as keyhole limpet hemocyanin (KLH) (Biosyn, Germany) and bovine serum albumin (BSA) (Sigma-Aldrich) a cysteine was added to either their N- or C-termini.

**Peptide Conjugation**

In order to chemically link the peptides to KLH or BSA the proteins were activated with the cross linker N-[γ-Maleimidobutyryloxy]succinimide ester (GMBS, Pierce) according to manufacturer’s instructions. Prior conjugation peptides were dissolved in 10% DMSO and then linked to activated KLH (50% of total volume) or BSA (10% of total volume) in 0.2 M Na-phosphate buffer, pH 6.8. The coupling efficiency was determined by Ellmann assay, measuring free SH groups and by HPLC-analysis before and after coupling.

**Peptide ELISA**

Blood plasma samples from vaccinated animals were tested for their binding to the original human PCSK9 sequence. For this purpose, 96-well Nunc MaxiSorp plates were coated with 1 µM of the appropriate peptide-BSA conjugate diluted in 0.1 M NaHCO_3_, pH 9.2-9.4. Free binding sites on the plates were blocked with blocking buffer (1% BSA in PBS, for 1 h at 37°C). Appropriate dilutions of the respective blood sample were added to the wells serially diluted 1:2 starting with a 1:400 dilution (dilution buffer: 1xPBS, 0.1% BSA, 0.1% Tween-20) and incubated for 1 h at 37°C. Each ELISA plate contained a standard antibody as internal control. For detection biotinylated anti-mouse IgG (H+L) (Southern Biotech. dilution 1:1000) was added for 1 h at 37°C, followed by horseradish peroxidase coupled to streptavidin for 30 min at 37°C. For visualization, the substrate ABTS (BioChemica, AppliChem) was added. After 30 min incubation at RT the optical density was measured at 405 nm with a Microwell plate reader (Sunrise, Tecan, Switzerland). The titers were defined as the plasma dilution factor at which 50% of the maximal optical density (ODmax) was reached.

**Protein ELISA**

To test the binding ability of the antibodies induced upon vaccination to the human PCSK9 (huPCSK9) protein a recombinant expressed human PCSK9 C-terminally fused to V5-His (“huPCSK9-V5-His”, AFFIRIS AG) was used. The amount of protein that was coated depended on the protein batch and was determined for each protein preparation before analyzing the sera to ensure comparable ELISA results. For IgE protein ELISA 2 µg/mL human IgE (NBS-C Bioscience) was coated.

**Gene Expression and Production of Human PCSK9-V5-His-Protein**

Briefly, PCSK9 cDNA sequence was subcloned from Origene TrueClone SC1011299 into the pcDNA3.1/V5-His-TOPO vector (Invitrogen, Life Technologies) and stably transfected into HEK293 cells for recombinant expression. Details of the procedure are available upon request.

**Plasma Levels of Murine PCSK9 (muPCSK9) and Target Engagement**

Plasma muPCSK9 concentration was determined by CircuLex mPCSK9 ELISA (CircuLex^TM^, Cy-8078, MBL, Woburn, MA) according to the manufacturer’s instructions. Briefly, 100 µL of the diluted 1:100 plasma sample was added on a 96-well microplate and incubated for 1 h. A HRP-conjugated anti-PCSK9 antibody was added for 1 h followed by the substrate reagent and stop solution. Optical density was detected at 450 nm with a Microwell plate reader (Sunrise, Tecan, Swizerland). PCSK9 concentration was defined using a standard curve provided by the supplier. The same kit CircuLex mPCSK9 ELISA was used for the target engagement analysis, but here detection with biotinylated anti-mouse IgG (H+L) (Southern Biotech; dilution 1:2000) for 1 h at 25°C was performed immediately after incubation, followed by the substrate reagent and stop solution provided by the supplier. The optical density was detected at 450 nm. The capture and the AFFITOPE^®^-generated antibodies differ from their epitope origin.

**LDLR Sandwich ELISA**

To determine the levels of LDLR in murine liver, 96-well Nunc MaxiSorp plates were coated with goat polyclonal anti-LDLR (R&D Systems, AF2255). Plates were blocked with 1% BSA in PBS. Liver lysates were incubated for 2 h at RT to capture the murine LDLR. The detection of captured LDLR was performed by rabbit anti-LDLR (Proteintech 10785-1-AP), followed by incubation with a secondary biotinylated goat anti-rabbit IgG (Southern Biotech, 4030-08) and by streptavidin-HRP conjugate (Roche). Finally, TMB (Sigma-Aldrich) was used as a peroxidase chromogen substrate, and the reaction was stopped using stop reagent (Sigma-Aldrich) and measured at 450 nm. The quantification of LDLR was made by comparison to a standard calibration curve performed with recombinant mouse LDLR (R&D Systems, 2255-LD) and was normalized to the total protein concentration of the lysates measured by BCA (Pierce). The negative control group was set to 100%, and used as a reference.

**Affinity Determination**

Affinity parameters of vaccine-induced antibodies were analyzed by surface plasmon resonance (SPR) (BiaCore^®^). Biotin-tagged antigen (native PCSK9 epitope) (EMC, Tübingen, Germany) was immobilized on the surface of a streptavidin-coated BiaCore^®^-sensor chip using HEPES-buffered saline, pH 7.4 (HBS) as running buffer. Approximately 100 response units (RU) of the peptide were loaded on the chip, flow cell 1 was left empty and used as a reference (background signal). Subsequently, free streptavidin binding sites were blocked with free biotin (Sigma-Aldrich) and naϊve serum (1:100). 100 µl of each plasma sample (dilution 1:100 in HBS) at a flow rate of 30 µl/min were injected and chip surface was regenerated with 15 µl of 10 mM glycine, pH 2.6 after each plasma injection. After each run the background signal of the flow cell 1 was subtracted from the signals obtained by the ligand-bound flow cells. The stability of the chip-surface was controlled by repeated injections of control antibody. For evaluation RU values at the end of serum injection were used as an indicator for the total amount of the bound antibodies. Off-rate values (1/s) were calculated using the BIA evaluation software (1:1 Langmuir interaction model for dissociation). The off-rate describes the dissociation velocity of the antibodies from the ligand and constitutes, beside on-rate, the second relevant parameter for affinity determination derived from individual serum samples.

**Inhibition of huPCSK9-huLDLR Interaction**

The ability of the AFFITOPE^®^-induced antibodies to inhibit the PCSK9-LDLR interaction was analyzed by SPR. Briefly, huLDLR (R&D Systems) was immobilized via amine coupling to a CM5 sensor chip. Plasma samples from immunized mice and non-specific (negative) controls were pre-incubated for 2 h at RT with and without huPCSK9-V5-His (AFFiRiS AG) (1:10 dilution in degassed Tris buffer (20 mM Tris-HCl, 100 mM NaCl, 1 mM CaCl_2_, 1% BSA, 0.1% Tween-20, pH 6.6). Consecutive samples were passed over the sensor chip surface and binding of the huPCSK9 to huLDLR was measured in response units (RU). Finally, the binding of the huPCSK9 incubated with non-specific (negative) control samples was set as 100% and used as a reference.

**Fast Protein Liquid Chromatography (FPLC) Cholesterol Profile**

FPLC cholesterol lipoprotein profile of pooled plasma samples was analyzed at the Lipid and Lipid Metabolite Analysis Core Facility, Katz Group Centre for Pharmacy and Health Research, Department of Pediatrics, University of Alberta. The analyses were performed according to their protocols. Briefly, pooled plasma samples were separated on a Superose 6 HR 10/30 column, and the detection of the absorbance units (mAU) was performed at 500 nm.

**T cell Epitope Prediction**

*In silico* analysis for MHC class I and II binders was performed using the amino acid sequence of Peptide #1 (peptide antigen in the anti-PCSK9 vaccine). For this analysis the online database SYFPEITHI was used (Tübingen, Germany, <http://www.syfpeithi.de>). In addition, potential T cell epitopes were also identified using the Immune Epitope Database and Analysis Resource (IEDB-AR) (http://tools. immuneepitope.org/analyze/html/mhc_binding.html). Here T cell epitopes were classified based on their binding affinity for major histocompatibility complex (MHC) alleles, using the half-maximal inhibitory concentration of a biological substance (IC_50_) as unit of measure.

**Detection of T cell Activation using ELISpot**

Four 8-10 week-old mice per group were immunized 4 times in a biweekly interval with the indicated vaccines. Mice were sacrificed 5-7 days after their last immunization. Splenocytes were isolated from each individual mouse and cell pools within each group were prepared. Subsequently, red blood cells were lysed using ACK buffer (0.15 M NH_4_Cl, 1.0 M KHCO_3_, 0.1 mM Na_2_EDTA). Splenocytes were then washed, passed through a 70 µm cell strainer (BD Falcon) and counted using trypan blue (Gibco). The mouse INF-γ cytokine assay (Mouse INF-γ ELISpot^PLUS^ Kit (ALP), Mabtech) was used according to the manufacture instructions. Briefly, wells were coated with 15 µg/mL AN18 (anti-INF-γ) antibody (Mabtech) diluted in PBS, by incubation overnight at 4°C. Plates were then washed 5 times with PBS prior to incubation with 200 µl RPMI medium (Gibco) supplemented with 10% Fetal Calf Serum (FCS) (Gibco) and 1% Penicillin (1U/mL)/Streptomycin (100µg/mL) (P/S) (Gibco) for at least 30 min at RT to block non-specific binding. After blocking, splenocytes were added at 5 x 10^5^ cells per well and re-stimulated either with 10 µg/mL peptide (EMC, Tuebingen), 20 µg/mL PHA (Phytohemagglutinin) (Sigma-Aldrich), 10 µg/mL PCSK9-V5-His (AFFIRIS AG) or RPMI medium (Gibco) supplemented with 10% FCS (Gibco) and 1% P/S (Gibco) and incubated for 20 h at 37°C. Detection was performed according to the manufacturer’s guidelines, and the amount of INF-γ producing cells were determined by counting the number of spots per well using an AID EliSpot Reader (Software ELISPOT 3.5). Results were expressed as the number of anti-INF-γ spot-forming cells (SFC) per 5 x 10^5^ cells plated. Triplicate measurements were performed for each group.

**Protein Basic Local Alignment Search Tool (BLAST)**

*In silico* sequence homology analysis of the selected peptide with other human proteins was evaluated by using the protein BLAST [1] platform (<http://www.ncbi.nlm.nih.gov/BLAST/Blast.cgi>). Briefly, the selected sequence was BLASTed for sequence homology with human proteins available in the protein database. To evaluate the cross-reactivity of elicited anti-PCSK9 antibodies, BLAST search hits were further analyzed by direct ELISA.

**Antibody Purification**

Vaccine-induced antibodies were evaluated for possible cross-reactivity to human proteins using ProtoArray^®^ (Invitrogen, Life Technologies). Anti-PCSK9 antibodies were purified from mouse plasma after the last immunization using peptide specifically coupled to magnetic beads (Bioclone). The purification was performed according to the manufacturer’s guidelines. Briefly, prior to coupling, beads were washed three times with coupling buffer (50 mM Tris-HCl, 5 mM EDTA, pH 8.5) followed by coupling in coupling buffer (50 µg peptide to 5 mg beads). Subsequently, blocking of the unbound active groups on the beads was performed by incubating with coupling buffer containing 8mg/ml L-Cysteine•HCl (60 min at RT) by gentle rotation. To purify antibodies, 50 µl mouse plasma was incubated (2 h, RT) under constant agitation. Finally, elution has been performed in elution buffer (0.2 M glycine, 0.15 M NaCl, pH 1.9); purified antibodies were stored at 4°C.

**Immunoprecipitation of Plasma Antibodies**

The immunoprecipitation (IP) was performed according to the manufacturer’s guidelines. Briefly, prior to immunoprecipitation, GammaBind G Sepharose beads (GE Healthcare) were washed five times with 1x PBS, followed by pre-incubation of the plasma samples (diluted 1:4) with GammaBind G Sepharose for 2 h at 4°C (with rocking), Subsequently, the supernatant was transferred to new GammaBind G Sepharose beads followed by incubation overnight at 4°C (with rocking). Finally, detection of antibodies directly bound to PCSK9 was performed in samples (diluted 1:4) prior and post immunoprecipitation.

**Human Protein Array Analysis**

Anti-PCSK9 antibodies generated upon anti-PCSK9 immunization were affinity purified and evaluated for their specificity by cross-reactivity analysis to other human proteins using the ProtoArray^®^ platform (Invitrogen, Life Technologies). The detection was performed according to the manufacturer’s guideline with 1µg/mL goat anti-mouse IgG Alexa Fluor 647 (Invitrogen, Life Technologies) and for scanning of the array the molecular device GenPix 400B at wavelength 635nm was used. Finally, ProtoArray^®^ microarray results were evaluated by the software ProtoArray^®^  Prospector v5.2 according to the guidelines.

**Double Immunofluorescence Analysis of Frozen Human Tissue**

Frozen Human Tissue Panels (BioChain^®^) were warmed up to room temperature for 30 min and prior to immunostaining fixed for 10 min in Roti^®^-Histofix 4% (Roth). As primary antibodies α-MFAP1 (Novus Biological) and affinity purified Peptide #1-generated anti-PCSK9 antibodies were used. Each tissue panel was treated with secondary antibodies only as controls. Immunostaining of anti-MFAP1 antibody and affinity purified anti-PCSK9 antibodies was visualized using appropriate secondary antibodies conjugated with Alexa 488 or 594 (Invitrogen, Life Technologies) followed by counterstaining by VECTASHIELD^®^ HardSet^TM^ Mounting Medium with DAPI (Vector Laboratories). Epifluorescence was performed at room temperature using Immersol 518 (Carl Zeiss, Inc.) as an imaging medium and a microscope (AxioImager M2; Carl Zeiss, Inc.) equipped with an AxioCam MRm camera (Carl Zeiss, Inc.). Images were acquired using the ZEN pro 2011 software, Blue Edition (Carl Zeiss, Inc.).

**Modified SHIRPA Test**

The general health and neurological function of the mice immunized with anti-PCSK9 were evaluated by the well-known comprehensive SHIRPA test battery: a tool by which a broad screen of the mouse phenotype is obtained [2]. After the last immunization, the control group (injected with an irrelevant peptide) and anti-PCSK9 immunized mice were evaluated for their general health status, sensory and motor functions. General health functions included evaluation of the body weight, eye closure, lacrimation, salivation, piloerection, coat appearance, missing whiskers, tail curl, respiratory function and involuntary movements. The evaluation for sensory abilities included tests of front approach, abdominal reflex, eyelid reflex, tail pinch, visual forepaw reach, auditory startle, ear and whisker twitch. The motor abilities were tested by evaluation of body position, gait, movement, pelvic elevation, truck curl, righting reflex, clasping, forelimb grip and grasping reflex, and finally the test for the handling included vocalization and touch escape. Detailed information of the SHIRPA score evaluation is available online at the EMPReSS (European Mouse Phenotyping Resource Standardized Screens) database (<http://empress.har.mrc.ac.uk/>). The SHIRPA test was performed in an isolated experimental room at the mouse facility of the Institute of Molecular Biotechnology of Austrian Academy of Science (IMBA), Vienna, Austria.

**References**

1. Altschul SF, Gish W, Miller W, Myers EW, Lipman DJ (1990) Basic local alignment search tool. J Mol Biol 215: 403-410.

2. Brooks SP, Dunnett SB (2009) Tests to assess motor phenotype in mice: a user's guide. Nat Rev Neurosci 10: 519-529.
